# Insights into the Regulation of Indigo Production in an Engineered *Escherichia coli* Strain via Overexpression of Specific Transporter Genes and Proteomic Analyzes

**DOI:** 10.3390/foods15081385

**Published:** 2026-04-16

**Authors:** Jie Gao, Anni Fang, Tianjiao Meng, Baoguo Sun, Lei Cheng

**Affiliations:** Key Laboratory of Plant Protein Innovation and Resource Development, China National Light Industry, Beijing Engineering and Technology Research Center of Food Additives, Beijing Technology and Business University, Beijing 100048, China; jiegao0220@163.com (J.G.); ni1363785062@163.com (A.F.); mtianjiao0716@163.com (T.M.); sunbg@btbu.edu.cn (B.S.)

**Keywords:** indigo pigment, transport system, biosynthesis, anabolism, DIA-based quantitative proteomics

## Abstract

Conventional extraction of indigo, a vital natural dye, provides low yields and has a negative environmental impact. However, microbial synthesis has emerged as a sustainable alternative. In this study, we describe the optimization of indigo biosynthesis in an engineered *Escherichia coli* strain called E216. This strain carries, on a replicative plasmid, the *styAB* genes originating from *Pseudomonas putida* that constitute the monooxygenase biosynthetic pathway of indigo, as well as *mdh,* encoding malate dehydrogenase, which plays a role in reducing power generation. In this strain, the overexpression of *mtr* (a gene encoding a transporter of tryptophan (Trp), the precursor of indigo biosynthesis) and *acrA* (a gene encoding a protein involved in indigo efflux) was found to substantially enhance indigo yields. Consistently, knocking out these two genes using CRISPR-Cas9 significantly reduced indigo production, whereas it was restored through the complementation of these mutants. This study thus revealed that stimulating tryptophan uptake and indigo efflux, the latter of which limits indigo’s toxic intracellular accumulation, has a positive impact on indigo yields. Furthermore, a comparative mass spectrometry-based proteomic analysis of E216 grown in fermentation medium with or without tryptophan supplementation, integrated with data-independent acquisition (DIA), revealed the global impact of tryptophan supplementation on cellular metabolism. This analysis identified upregulation of key proteins and enriched metabolic pathways under conditions of tryptophan supplementation. Integrating the results of the genetic engineering and proteomic analysis establishes a strong scientific and practical basis for developing a highly efficient method for the green industrial production of indigo using engineered *E. coli* strains.

## 1. Introduction

Indigo, a deep blue crystalline powder used since antiquity, is among the world’s oldest natural dyes [1]. Today, the textile industry consumes large quantities of indigo each year for processes such as denim dyeing [2]. In addition, synthetic derivatives, particularly indigo carmine (E132), are employed as food colorants in products such as fruit juices, tomato ketchup, and bread [3,4,5]. Expanding applications of indigo—including in organic semiconductors, paints, and printing—have driven its annual production capacity to 70,000–80,000 tons [6]. Traditionally, indigo has been extracted from plants such as mullein, Polygonum species, and Radix Isatidis [7,8]. However, natural indigo extraction is hampered by low yields, cultivation constraints (low water availability, poor soil conditions, and susceptibility to natural disasters), and generally poor productivity [9,10]. While chemical synthesis has emerged as a more economical production route, chemical processes typically require harsh conditions—high temperatures and pressures as well as strong acids or bases—rendering them hazardous, laborious, and environmentally damaging [11]. For these reasons, microbial synthesis has attracted substantial research interest as a greener, more sustainable alternative [12,13].

**Figure 1:**
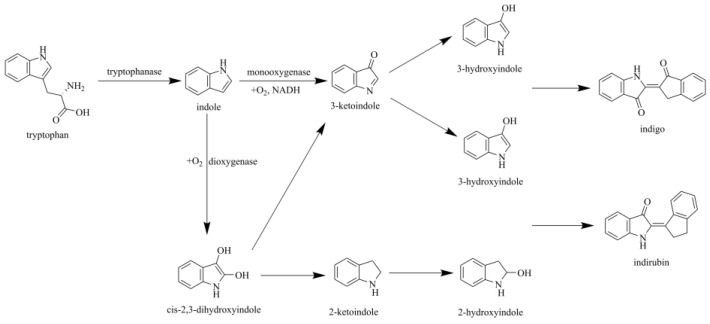
Indigo synthesis pathways.

As illustrated in Figure 1, under catalysis by tryptophanase, tryptophan is converted to indole, which serves as a key intermediate in the oxygenation reaction to form indigo. The conversion of indole to indigo is an oxygenation reaction catalyzed by two primary enzyme types: styrene dioxygenase and styrene monooxygenase. In the dioxygenase pathway, indole is converted into cis-2,3-dihydroxyindole, which is subsequently dehydrogenated and degraded to yield 2-oxoindole and 3-oxoindole. 2-Oxoindole isomerizes to 2-hydroxyindole, while 3-oxoindole isomerizes into two molecules of 3-hydroxyindole. These intermediates then condense to form indirubin, and two molecules of 3-hydroxyindole undergo further condensation to generate indigo [14]. Conversely, the monooxygenase pathway specifically incorporates a single oxygen atom into indole, directly producing 3-oxoindole. This intermediate isomerizes to yield two molecules of 3-hydroxyindole, which condense to form indigo [15,16]. Among these pathways, the styrene monooxygenase route demonstrates greater research potential due to its lower by-product generation.

*Escherichia coli* is a widely used microorganism for recombinant protein production [17,18] due to its rapid growth, ease of manipulation, and low cost, making it a reference in industrial fermentation and genetic engineering [19]. For indigo biosynthesis, efficient uptake of the substrate tryptophan is critical. In *E. coli*, three proteins—TnaB, Mtr, and AroP—are involved in the uptake of extracellular tryptophan [20]. Additionally, the efflux system is vital for indigo accumulation [21]. *E. coli* employs seven Resistance–Nodulation–Division (RND) transporters to expel harmful substances [22], six of which are involved in drug efflux. Optimizing these systems can significantly boost indigo production. However, current engineered strains still struggle with low substrate utilization and growth inhibition caused by intracellular indigo accumulation, limiting their application in the food industry.

Gene-editing tools—particularly CRISPR-Cas9—have recently enabled rapid optimization of microbial metabolic pathways. Derived from a bacterial adaptive immune system composed of Clustered Regularly Interspaced Short Palindromic Repeats (CRISPR) and CRISPR-associated (Cas) proteins, CRISPR-Cas9 targets and cleaves invasive DNA and is widely used for genome editing in *E. coli* [23]. Compared with conventional knockout methods, CRISPR-Cas9 is simpler, cheaper, more specific, and more efficient, and it enables multiplexed or sequential gene edits [24,25], facilitating complex metabolic engineering.

In our preliminary study, the indigo-producing *Pseudomonas putida* strain B3 was isolated, and indigo synthesis was achieved through heterologous expression of the styrene monooxygenase gene *styA* in *E. coli* BL21 using the pET-28a(+) plasmid. Subsequently, *styA* and *mdh,* a gene encoding a malate dehydrogenase that generates reducing power, were co-expressed under the control of a modified strong constitutive promoter. Through promoter engineering, five engineered strains (E212, E213, E214, E215, E216) overexpressing *styAB* and *mdh* under promoters of varying strengths were constructed [26]. The engineered strains were screened, and E212 and E216 (both originating from modified *E. coli* BL21), which showed the highest indigo production under identical conditions, were selected. Since indigo production yields are known to be limited by inefficient substrate/tryptophan uptake as well as by intracellular indigo accumulation that imposes a metabolic burden, genes encoding proteins involved in tryptophan uptake and indigo efflux were identified through the analysis of NCBI database and PCR validation of the identified genes. This analysis revealed 3 tryptophan permease encoding genes and 7 indigo efflux genes and transcriptomic analysis of E212 and E216 during fermentation identified *mtr* (tryptophan transport) and *acrA* (indigo efflux) as the most highly expressed genes. *mtr* and *acrA* were thus overexpressed or deleted in the E216 strain to investigate their roles in tryptophan transport and indigo efflux with the aim of enhancing indigo synthesis and thus supporting greener indigo production. Indeed, although the molecular targets of indigo toxicity remain poorly characterized, oxidative stress and membrane disruption may contribute to its cytotoxic effects [27]. Therefore, optimizing tryptophan transport into cells and indigo efflux is critical.

To complement gene-level engineering, proteomics was used to elucidate how tryptophan supplementation reshapes *E. coli*’s metabolic network. High-throughput proteomics quantitatively detects differential protein expression, overcoming the limits of single-gene analyses [28,29]. Data-Independent Acquisition (DIA) on an Orbitrap platform was adopted—a label-free, comprehensive quantitative strategy that does not require preselected peptides and acquires global data through fixed scanning windows. Coupled with a spectral library, DIA enables accurate peptide identification, retrospective data mining, high reproducibility, and robust quantitative performance [30]. Using DIA, the proteomes of strain E216 with and without tryptophan were compared to identify proteins and pathways associated with indigo synthesis; this approach both validates the genetic targets and illuminates the protein-level mechanisms by which tryptophan enhances production, providing insights for multi-omics metabolic engineering. This study aims to clarify the roles of the tryptophan permease *mtr* and the efflux gene *acrA* in indigo biosynthesis and to build an efficient production system based on substrate import and product export. By enhancing *mtr* to improve tryptophan uptake, modifying *acrA* to promote indigo excretion, and overexpressing key biosynthetic genes under the P_T7_ promoter, a significant increase in indigo yield was achieved. Proteomic validation via DIA confirmed these effects and helped map the underlying pathways, enabling a systematic evaluation of transport systems and optimization of the synthetic pathway.

The innovation of this research lies in the efficient expression of the indigo efflux gene *acrA* and the tryptophan permease gene *mtr*, combined with promoter engineering and the integration of proteomics, achieving multi-dimensional enhancement of indigo production capacity. Proteomic data confirmed that tryptophan supplementation significantly activates the expression of proteins such as AcrA and Mtr, which complements genetic overexpression. Furthermore, through differential protein enrichment analysis, the mechanism underlying the increased yield was elucidated at the molecular level. This multi-omics strategy not only enhances the engineered strain’s utilization of the substrate tryptophan but also improves indigo efflux efficiency, offering a novel perspective for constructing green and efficient *E. coli* strains for indigo production. This high-efficiency indigo production system is capable of reducing costs and increasing yields in industrial-scale manufacturing while aligning with the requirements of sustainable development and environmental friendliness, and thus holds significant economic and ecological importance. By integrating optimization at the genetic and protein levels to enhance *E. coli*’s indigo production mechanism, this research provides valuable theoretical support and a practical foundation for the field of industrial biotechnology.

Current strategies for enhancing microbial indigo biosynthesis primarily focus on pathway enzymes and cofactor regeneration, while the critical roles of substrate uptake and product export were largely overlooked. Insufficient tryptophan transport limits precursor supply, and intracellular indigo accumulation imposes a metabolic burden and inhibits growth. The literature lacks systematic investigations targeting the tryptophan permease gene *mtr* and the efflux pump gene *acrA*. To address this gap, this study combines targeted engineering of *mtr* and *acrA* with quantitative proteomic analysis to establish a more comprehensive optimization framework for indigo production in engineered *Escherichia coli*.

## 2. Materials and Methods

### 2.1. Enzymes and Chemicals

The indigo standard products tryptophan, N,N-dimethylformamide, and kanamycin were purchased from Sigma–Aldrich (St. Louis, MO, USA). PrimeSTAR Max Premix (2×) DNA polymerase, molecular quality standards for DNA Markers, and a 10,000 DL DNA Marker were obtained from Takara Bio (Dalian, China). Glycerol, bromophenol blue, SDS, urea, DTT, and IAA were sourced from Sangon Biotech, Bio-Rad Laboratories, and Merck, respectively. C18 trap and analytical columns were acquired from Thermo Fisher Scientific, while 5 kDa ultrafiltration tubes and solid-phase extraction MCX columns were acquired from Sartorius and Omics Solutions, respectively, and trypsin was purchased from Promega Corporation. Reagents used for buffer preparation included SDC lysis buffer (5% SDC, 100 mM Tris-HCl pH 8.5), UA buffer (8 M urea, 150 mM Tris-HCl, pH 8.0), and HPLC mobile phases (A: 0.1% formic acid; B: 0.1% formic acid, 80% acetonitrile). All other reagents were available on the domestic market and of analytically pure grade.

### 2.2. Bacterial Strains, Plasmids, and Culture Conditions

The bacterial strains and plasmids used in this study are listed in Table 1, while the primers used are listed in Table S1. *Pseudomonas putida* B3 was cultured at 30 °C, and *E. coli* strains were cultured in Luria–Bertani (LB) medium at 37 °C with vigorous shaking. The antibiotics spectinomycin, chloramphenicol, and kanamycin were added at a concentration of 50 µg/mL when needed.

### 2.3. Knockout of the Target Gene by CRISPR-Cas9 Technology

After thawing the *E. coli* BL21 cells, 2 μL of pCas9 plasmid was added, and the cells were subjected to heat shock at 42 °C for 1 min, followed by incubation on ice for 2 min. Subsequently, 900 μL of LB medium was added, and the cells were incubated at 37 °C with shaking (200 r/min) for 45 min. The transformed cells (100 μL) were plated onto chloramphenicol-containing agar and incubated at 37 °C for 12–16 h. Single colonies were inoculated into LB medium containing chloramphenicol to generate the BL21-Cas9 strain. For overnight culture, a single colony was inoculated into LB medium with chloramphenicol and grown at 37 °C and 200 r/min. The next day, the culture was diluted 1:100 into fresh LB medium with chloramphenicol and grown until the OD600 reached 0.2–0.3. Arabinose was then added to induce gene expression until the OD600 reached 0.5–0.6. For receptor cell preparation, the culture was cooled and centrifuged, and the pellet was resuspended multiple times with pre-cooled sterile water.

The N20 sequences targeting the *acrA* and *mtr* genes were designed using the CRISPR targeting design tool [30]. Primers were constructed based on the N20 sequences, and the N20 sequences were inserted between the promoter and the gRNA scaffold of the pTargetF plasmid via PCR amplification. The primers used are listed in Table 1. The PCR cycling conditions were as follows: 98 °C for 5 min; 30 cycles of 98 °C for 10 s, 55 °C for 5 s, and 72 °C for 11 s; and a final extension at 72 °C for 10 min. The product was analyzed via 1% agarose gel electrophoresis, and the target band was excised and purified using a Universal DNA Purification Kit (Tiangen Biotech, Beijing, China). The knockout recombinant plasmid pTargetF was then constructed using the ClonExpress II One Step Cloning Kit C112 (Vazyme, Nanjing, China). To achieve this, linearized vector fragments were prepared via PCR using PrimeSTAR Max (2X) Premix (Tiangen Biotech, Beijing, China) under the following conditions: 98 °C for 5 min; 30 cycles of 98 °C for 10 s, 55 °C for 5 s, and 72 °C for 9 s; and a final extension at 72 °C for 10 min. The primers used are listed in Table S1. Homology arms were added to the target gene fragments via PCR using *E. coli* BL21 genomic DNA (extracted with a Bacterial Genomic DNA Extraction Kit, Tiangen Biotech, Beijing, China) as the template. The PCR conditions were as follows: 98 °C for 5 min; 30 cycles of 98 °C for 10 s, 55 °C for 5 s, and 72 °C for 4 s; and a final extension at 72 °C for 10 min. The pTargetF recombinant plasmid was then electroporated into BL21-Cas9 receptor cells, generating knockout strains designated as the *acrA*-BL21 and *mtr*-BL21 series.

The constructed strains were subjected to quantitative fluorescence detection and colony PCR. Colonies showing no detectable bands for the target genes were selected and named BL21-*acrA* and BL21-*mtr*. To further verify knockout success, primers located 500 bp upstream and downstream of the target genes were used for amplification under the following cycling conditions: 98 °C for 5 min; 30 cycles of 98 °C for 10 s, 55 °C for 5 s, and 72 °C for 5 s/kb; and a final extension at 72 °C for 10 min. Strain E216 was cultured, and the plasmid pETPcat-*styAB*-P_T7_-*mdh* was extracted using a High Pure Plasmid Mini Preparation Kit (Tiangen Biotech, Beijing, China). The plasmids were then electroporated into BL21-*acrA* and BL21-*mtr* receptor cells, yielding the *acrA* knockdown E11 and *mtr* knockdown E21, respectively.

### 2.4. Engineering Bacteria Using acrA and mtr Gene Complementation and Overexpression

Genomic DNA was extracted from BL21 cells using the Bacterial Genomic DNA Extraction Kit (Tiangen Biotech, Beijing, China) and diluted to 30 μg/mL for PCR amplification of the *acrA* and *mtr* genes. The PCR products, incorporating a T7 promoter, were cloned using the ClonExpress II One Step Cloning Kit C112 to generate pETPcat-*styAB*-P_T7_-*mdh*-*acrA*/*mtr* and pETPcat-*styAB*-P_T7_-*mdh*-T7-*acrA* fragments, which were subsequently quantified. Following extraction of the E216 plasmid, these fragments were used to construct recombinant plasmids. By transforming pETPcat-*styAB*-P_T7_-*mdh*-*acrA* into E11 and E216, the back-complemented bacterium E12 and the *mtr* overexpression strain E23 were obtained. Similarly, introducing pETPcat-*styAB*-P_T7_-*mdh*-*acrA*/*mtr* into E216 yielded E13 and E14, while introducing pETPcat-*styAB*-P_T7_-*mdh*-T7-*acrA*/*mtr* into E216 produced E22, E23, and E24.

### 2.5. Indigo Production Assay for Each Engineered Bacterium

The fermentation medium used for indigo production contained the following components per liter: 18.0 g Na_2_HPO_4_, 3.5 g yeast extract, 3.2 g KH_2_PO_4_, 1.2 g NH_4_Cl, 0.7 g NaCl, and 0.2 g MgSO_4_. The medium was sterilized through autoclaving at 121.0 °C for 17.0 min. After cooling to room temperature, tryptophan was added to a final concentration of 0.1 mg/mL.

In a laminar flow hood, each modified bacterium was inoculated into 5 mL LB medium containing 5.0 μg/mL kanamycin and incubated with shaking at 37 °C and 200 r/min for 12 h. After activation, 0.6 mL of the culture was added to 50 mL of fermentation medium and incubated at 30 °C, 200 r/min, and pH 7.0 for 24 h to produce indigo fermentation broth. The broth was centrifuged at 9000 r/min for 25 min, and the supernatant was discarded. The pellet was resuspended in N,N-dimethylformamide and ultrasonicated for 30.0 min to disrupt the cells and release indigo. An indigo standard was dissolved in N,N-dimethylformamide to prepare a 50.0 μg/mL stock solution, which was then diluted to create standard solutions of 7.0, 9.0, 11.0, 13.0, and 15.0 μg/mL. The absorbance of each standard solution was measured at 610 nm using a spectrophotometer, and a standard curve was constructed. Finally, the absorbance of the indigo extract was measured at 610 nm, and the concentration was determined from the standard curve.

### 2.6. Growth Curve Determination

To investigate the growth dynamics of each modified bacterium in the fermentation medium, plate counting was employed to determine the total cell count. The seed culture was inoculated into the fermentation medium, and aliquots were collected at 4 h intervals. These samples were diluted 10^6^- or 10^7^-fold, spread onto solid fermentation media, and incubated at 37 °C for 24 h. Each dilution was tested in triplicate. Following colony counting, the growth curves were plotted, with sampling time along the *x*-axis and colony counts along the *y*-axis.

### 2.7. Proteomics Experimental Design and Methodology

#### 2.7.1. Experimental Design and Sample Preparation

The high indigo-yielding engineered strain E216 was used as the parental strain. An experimental group (supplemented with a final concentration of 0.1 g/L tryptophan) and a control group (without tryptophan) were established, each comprising three biological replicates, to assess the impact of tryptophan on global protein expression. Samples were collected at the mid-logarithmic growth phase (OD600 = 0.6) to ensure stable metabolic activity. Immediately after collection, cells were washed with pre-cooled PBS buffer to remove medium impurities.

#### 2.7.2. Protein Extraction and Quantification

SDC lysis buffer (5% SDC, 100 mM Tris-HCl pH 8.5) was added to the cell pellet, vortexed thoroughly, and boiled for 15 min to achieve complete cell lysis. The lysate was centrifuged at 4 °C, 14,000× *g* for 40 min, and the supernatant was collected as the protein extract. The protein concentration of the extract was determined using a BCA protein quantification kit (Beyotime, Shanghai, China). For each sample, 15 µg of protein was taken, mixed with 5× loading buffer, and boiled for 5 min for subsequent analysis.

#### 2.7.3. Protein Separation and Enzymatic Digestion

Protein samples were separated using a 4–20% gradient SDS-PAGE gel (constant voltage of 180 V for 45 min), and stained with Coomassie Brilliant Blue R-250 to confirm extraction quality. Subsequently, gel bands were treated with dithiothreitol (DTT, reduction at 37 °C for 1.5 h) and iodoacetamide (IAA, alkylation at room temperature in the dark for 30 min) to reduce disulfide bonds and alkylate cysteine residues. Trypsin (enzyme-to-protein mass ratio of 1:50) was added, followed by incubation at 37 °C overnight (15–18 h) to complete the enzymatic digestion of proteins.

#### 2.7.4. Peptide Desalting and Mass Spectrometry Data Acquisition

Digested peptides were desalted and purified using MCX (omicsolution; Suzhou, China; OS-MCX-1ML) solid-phase extraction columns, concentrated via vacuum centrifugation, and reconstituted in an aqueous solution containing 0.1% formic acid. Peptide concentration was estimated by UV spectrophotometry at 280 nm. To enhance mass spectrometry quantification accuracy, iRT calibration peptides were added as internal standards. Peptide samples were analyzed using a Vanquish Neo liquid chromatography system coupled with an Orbitrap Astral mass spectrometer, operating in data-independent acquisition (DIA) mode. The DIA parameters included a full scan range of 350–1500 m/z and fragmentation energy of 28 eV, ensuring high-throughput and unbiased global protein detection.

#### 2.7.5. Data Analysis and Functional Annotation

Raw mass spectrometry data from DIA mode were processed using DIA-NN software (version 1.8.1). The key parameters included the following: enzyme set to trypsin (max missed cleavages = 1); carbamidomethyl as (C) a fixed modification; and oxidation (M) and acetyl (Protein N-term) as dynamic modifications. Protein identification was based on a false discovery rate (FDR) ≤ 1% with at least two unique peptides using the R function hclust in R (version 4.2.0). The Euclidean distance algorithm for similarity measurement and the average linkage clustering algorithm (clustering uses the centroids of the observations) for clustering were selected for hierarchical clustering. Additionally, principal component analysis (PCA) was performed using the R function prcomp (R version 4.2.0) to assess data reproducibility and group separation, with Pareto scaling applied for normalization.

For functional annotation, the protein sequences of the selected differentially expressed proteins were first processed using the Blast2Go software (version 3.2) [31]. This involved a local BLAST search to identify homologues, followed by mapping and annotation with Gene Ontology (GO) terms. The resulting GO annotation data were visualized using R scripts, and the same set of proteins was then subjected to KEGG annotation by blasting them against the online Kyoto Encyclopedia of Genes and Genomes (KEGG) database [32], retrieving their KEGG orthology (KO) identifiers, and mapping them to corresponding metabolic and signaling pathways.

The protein–protein interaction (PPI) data for the investigated proteins were accessed via the STRING database (version 12.0) (https://string-db.org/, accessed on 25 February 2026) [33], with high-confidence interaction scores (≥0.700) employed to ensure reliability. The interaction network was visualized using the igraph package (version 1.3.4) in R (version 4.2.0). Furthermore, the degree of each protein was calculated to evaluate its importance in the PPI network.

## 3. Results

### 3.1. Impact of Gene Regulation on Indigo Production

#### 3.1.1. Effect of *acrA* Gene on Indigo Production

CRISPR-Cas9 technology was employed to disrupt the *acrA* gene. Five N20-targeting sequences were designed, and corresponding pTargetF plasmids were constructed to generate five knockout strains. Quantitative fluorescence analysis identified N20-4 as the most effective construct, so this strain was selected for further screening. Single colonies were isolated and used as templates for colony PCR. Primers flanking the *acrA* locus (500 bp upstream and downstream) failed to amplify a band, confirming the absence of the target gene. Subsequent sequencing verification confirmed the deletion of *acrA* in the genome, confirming successful knockout. The resulting strain was designated E11.

**Figure 2:**
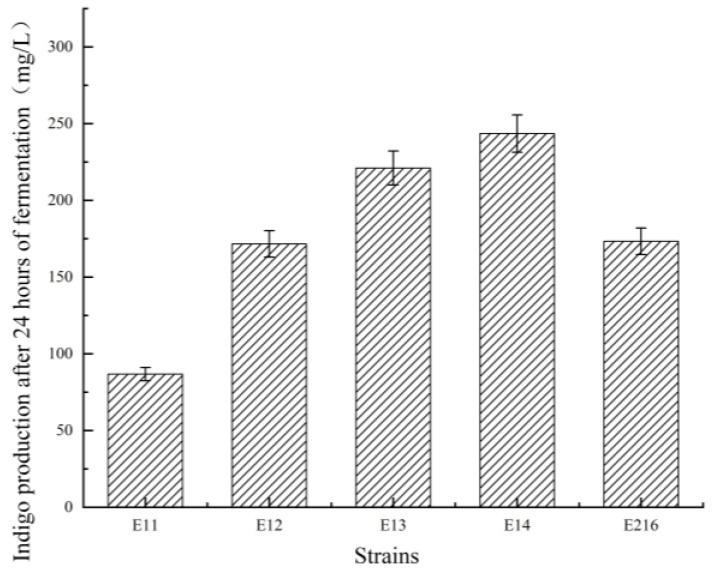
Comparison of indigo yields across engineered strains. E11: *acrA* knockout mutant; E12: E11 complemented with native *acrA*; E13 and E14: two independent *acrA*-overexpressing strains in which the expression of *acrA* was put under the control of the T7 promoter; E216: parental strain.

When inoculated into the fermentation medium, the E11 knockout strain exhibited significantly reduced indigo production compared to the parent strain E216. This decline indicates that the absence of the *acrA* gene inhibits the cellular transport of indigo and other compounds, leading to intracellular accumulation and premature cell death. To restore functionality, the *acrA* gene was reintroduced into E11 to construct the complemented strain E12. Following complementation, indigo production partially recovered, confirming the restoration of indigo transport capacity and alleviation of cellular burden, which in turn revitalized the cells and improved yields. Furthermore, the *acrA* overexpression strains E13 and E14, driven by the T7 promoter, showed significantly higher indigo production than the parent strain E216. The indigo yields of these engineered strains are presented in Figure 2.

These findings suggest that the AcrA protein, encoded by the *acrA* gene, facilitates indigo efflux from cells to the extracellular environment. By mitigating intracellular toxicity, this transporter enhances indigo synthesis efficiency. Furthermore, *acrA* overexpression significantly improved both indigo efflux and cellular vitality, offering substantial benefits for industrial-scale indigo production.

#### 3.1.2. *mtr* Gene Knockout and Its Effect on Indigo Production

**Figure 3:**
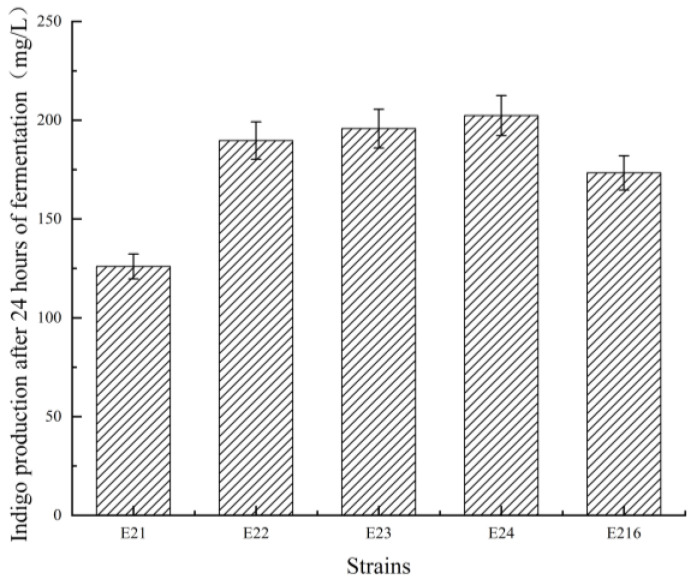
Comparison of indigo yields across engineered strains. E21: *mtr* knockout mutant; E22: E21 complemented with native *mtr*; E23 and E24: two independent mtr-overexpressing strains in which the expression of *mtr* was put under the control of the T7 promoter; E216: parental strain.

For the knockout of the *mtr* gene, a strategy analogous to that used for *acrA* was adopted. Five N20 sequences were designed, and quantitative fluorescence analysis identified N20-1 as the most efficient. Screening the colonies yielded a successful knockout strain, designated E21. Both genomic PCR amplification and sequencing confirmed the *mtr* gene disruption. Indigo production in the E21 knockout strain decreased significantly. To address this, the *mtr* gene was complemented in E21 to construct the complementary strain E22, which partially restored indigo yields. Furthermore, the overexpression strains E23 (constitutive) and E24 (T7 promoter-driven) were constructed. Indigo production in both overexpression strains was significantly higher than in the parent strain E216, as detailed in Figure 3. These findings suggest that the tryptophan transporter encoded by *mtr* facilitates the import of extracellular tryptophan, thereby supplying substrates for indigo synthesis. *mtr* knockout impaired tryptophan transport and indigo production, while its overexpression enhanced substrate utilization and increased yields. Specifically, the T7 promoter further elevated gene expression, leading to a significant boost in indigo synthesis capacity.

#### 3.1.3. Strain Growth Curve Determination

**Figure 4:**
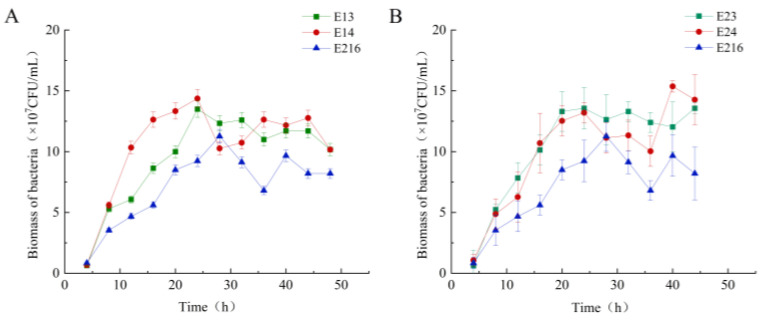
Growth curves of the parental strain and of the *mtr* and *acrA* overexpressing strains in the fermentation medium. (**A**) Growth curves of E216 (parental strain), E23 and E24 (two independent *mtr*-overexpressing strains in which the expression of *mtr* was put under the control of the T7 promoter; (**B**) Growth curves of E216 (parental strain), E13 and E14: two independent *acrA*-overexpressing strains in which the expression of *acrA* was put under the control of the T7 promoter.

To investigate the potential adverse effects of heterologous protein expression on host strains, growth curve assays were performed. Figure 4 presents the growth profiles of the *acrA*- and *mtr*-engineered strains in the fermentation medium. Both overexpression strains and the parental strain were inoculated, with samples plated every four hours to determine colony counts. As shown in Figure 4A, the E13 and E14 curves exhibited an initial rise followed by a decline and an eventual plateau, with the overall cell density exceeding that of the parental strain. This indicates that *acrA* overexpression enhances cell growth and vitality. Similarly, *mtr* overexpression strains (E23 and E24) demonstrated improved growth compared to the parental strain (Figure 4). E23 showed sustained growth until 25 h, plateauing from 25 to 45 h, with a slight decline thereafter. E24 displayed a biphasic pattern, increasing until 25 h, declining from 24 to 35 h, and recovering thereafter. These results demonstrate that *mtr* overexpression does not impair cellular vitality and may even enhance it.

#### 3.1.4. Effect of Fermentation Temperature on the Yield of Indigo

**Figure 5:**
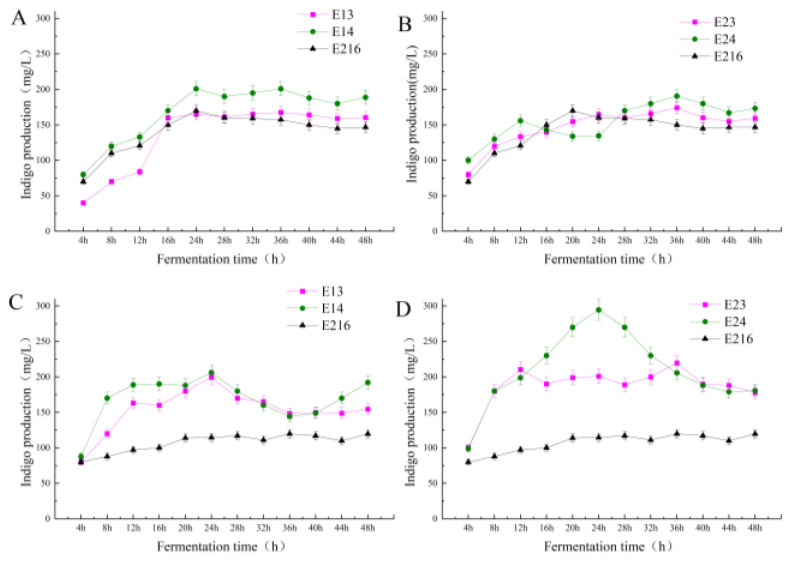
Production of indigo of the parental strain and of the *mtr* and *acrA* overexpressing strains throughout growth in the fermentation medium at different temperatures: (**A**) *acrA*-overexpressing strains E13 and E14 and parental strain E216 at 30 °C; (**B**) *mtr*-overexpressing strains E23 and E24 and parental strain E216 at 30 °C; (**C**) *acrA*-overexpressing strains E13 and E14 and parental strain E216 at 37 °C; (**D**) *mtr*-overexpressing strains E23 and E24 and parental strain E216 at 37 °C. Indigo yield is expressed as mg/L of culture volume.

Studying the effects of gene manipulation on indigo synthesis under varying temperature conditions revealed significant yield enhancements. As shown in Figure 5A, at 30 °C, the engineered strains E13 and E14 (*acrA*) produced higher yields than the parent strain E216. Indigo accumulation increased rapidly during the initial fermentation phase (0–24 h), temporarily dipped between 24 and 28 h, and then stabilized. E14 reached its peak yield of 200 mg/L at 24 h (with indigo yield expressed as mg/L of culture volume), likely due to the *acrA* gene’s efflux function. By facilitating timely indigo export, this gene alleviates cellular metabolic burden, enhances vitality, and boosts yields. Furthermore, the introduction of the T7 promoter further amplified this effect, further improving yields. Similarly, Figure 5B illustrates that at 30 °C, the *mtr*-engineered strains E23 and E24 outperformed the parent strain E216. The yield of E24 consistently exceeded that of E23, which in turn surpassed the control. Notably, E24 reached a peak yield of approximately 190 mg/L at 36 h. The analysis indicates that *mtr* overexpression significantly improved tryptophan transport efficiency, a mechanism that played a crucial role in enhancing indigo synthesis. Figure 5C demonstrates that at 37 °C, the *acrA*-engineered strains E13 and E14 maintained significantly higher yields than E216. E13 peaked at 206 mg/L at 24 h, followed by a brief decline before yields stabilized at 36 h. This fluctuation likely resulted from transient cellular inhibition due to excessive indigo accumulation, followed by active efflux that alleviated metabolic burden and restored stability. These results confirm that overexpression of the indigo efflux gene significantly enhances both cellular stability and production efficiency. Figure 5D shows that at 37 °C, the *mtr*-engineered strains, particularly E24, exhibited significant improvements. During the 4–24 h phase, E24 rapidly increased its yield, reaching a peak of 300 mg/L at 24 h—far surpassing the parent strain. This phenomenon is likely attributed to the *mtr*-encoded tryptophan transporter, which enhances indigo synthesis during the critical early fermentation phase at 37 °C. Although E23 showed more moderate yields, they still exceeded those of the control, indicating that *mtr* expression contributes to efficiency even without the T7 promoter.

### 3.2. Proteomic Analysis Results

To elucidate the global impact of tryptophan supplementation on *E. coli* metabolic networks, we conducted DIA proteomic analysis to compare the parental strain E216 with and without tryptophan supplementation (E216-1 vs. E216-0). Data quality validation demonstrated clear group separation via PCA, with tight intra-group clustering. Pearson correlation (>0.95) and QC sample coefficient of variation (median < 20%) confirmed excellent reproducibility and compliance with analytical standards. Leveraging this high-quality dataset, we performed a comprehensive analysis of differentially expressed proteins.

#### 3.2.1. Differentially Expressed Protein Analysis

**Figure 6:**
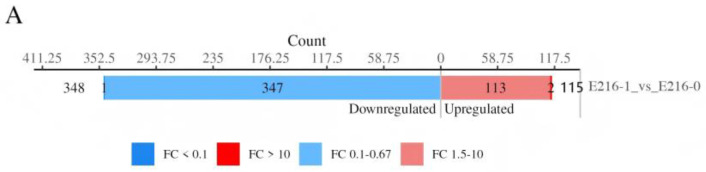

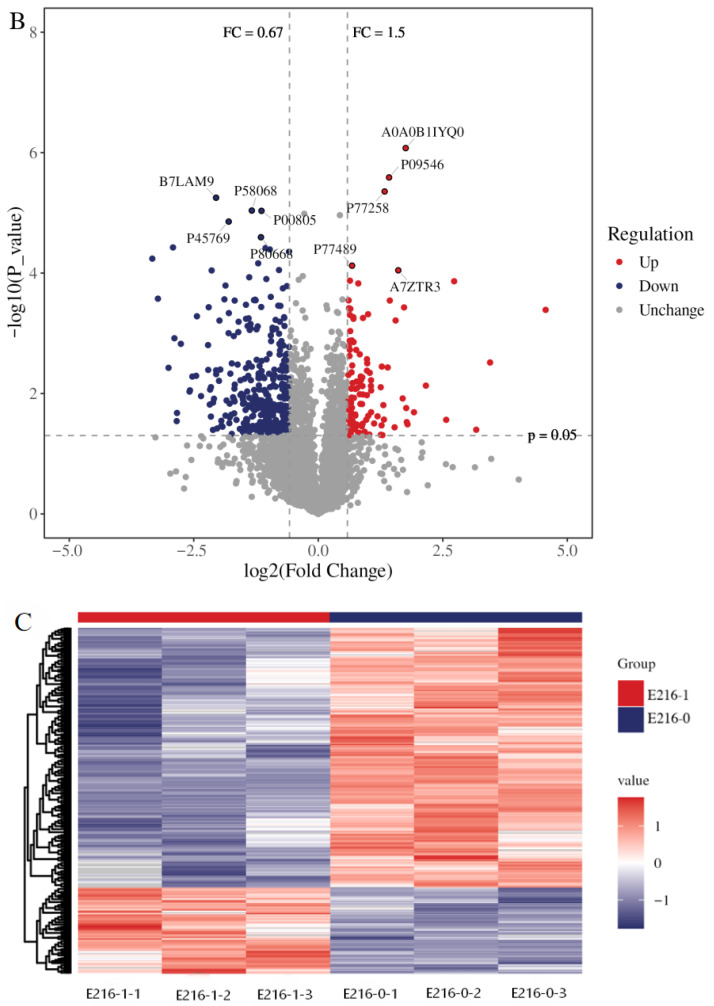
Analysis of differentially expressed proteins (DEPs) in E216-1 (tryptophan-supplemented) vs. E216-0 (control). (**A**) Number of upregulated (FC > 1.5, *p* < 0.05) and downregulated (FC < 0.67, *p* < 0.05) DEPs. (**B**) Volcano plot showing log2(fold change) vs. –log10(*p*-value). Red: upregulated; blue: downregulated; gray: unchanged. (**C**) Hierarchical clustering heatmap of DEPs across three biological replicates per condition. Euclidean distance and average linkage clustering were applied. The color scale represents Z-score-normalized protein abundance.

To identify differentially expressed proteins (DEPs), this study defined significance as fold change (FC) > 1.5 (upregulated) or FC < 0.67 (downregulated) with a *p*-value < 0.05 (*t*-test). These criteria filtered the data, revealing 113 upregulated and 348 downregulated DEPs in the E216-1 vs. E216-0 comparison (Figure 6A). Indicated by darker bars in Figure 6C, proteins with substantial fold changes provide a robust basis for subsequent functional analysis. A volcano plot (Figure 6B) visualizes this distribution: red dots indicate significant upregulation, blue dots denote downregulation, and gray dots represent unchanged proteins. Notably, downregulated proteins were predominant, with several exhibiting highly significant downregulation (log2(FC) < 2.5). Hierarchical clustering (Figure 6C) further illustrates the distinct protein expression profiles between E216-1 and E216-0. The clear separation, coupled with high consistency among biological replicates, underscores excellent experimental reproducibility.

#### 3.2.2. Functional Enrichment Analysis

**Figure 7:**
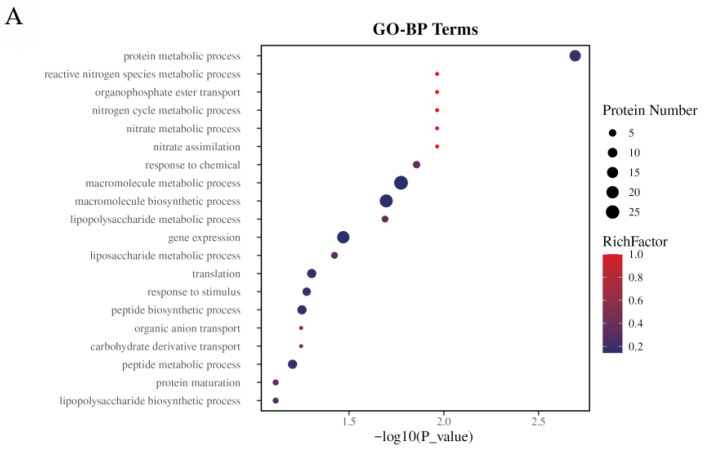

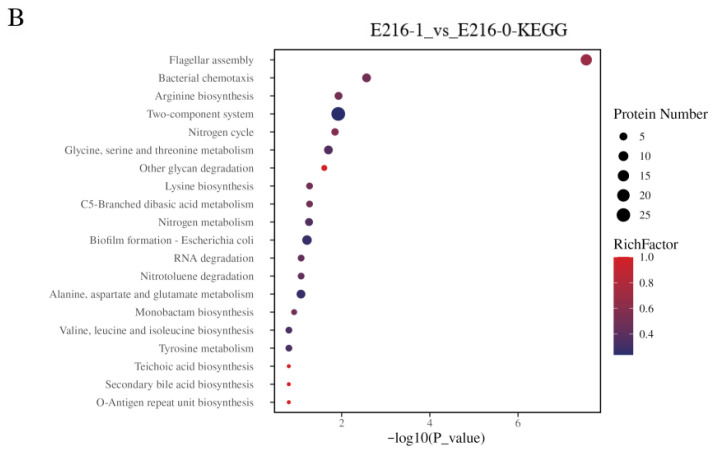
Functional enrichment analysis of DEPs in the E216-1 vs. E216-0 comparison. (**A**) Gene Ontology (GO) enrichment bubble plot showing significantly enriched biological processes (top 10). (**B**) KEGG pathway enrichment bubble plot showing significantly enriched pathways (top 10). Enrichment significance was assessed using a hypergeometric test with Benjamini–Hochberg correction (adjusted *p* < 0.05). Bubble size indicates gene count; color indicates adjusted *p*-value.

Functional enrichment analysis using GO terms (Figure 7A) indicated that tryptophan supplementation significantly enriched biological processes related to protein metabolism, reactive nitrogen species metabolism, and organophosphate ester transport. These results indicate how *E. coli* rewires its metabolism upon tryptophan supplementation and show that this rewiring is beneficial for indigo biosynthesis. First, enrichment in protein metabolic processes implies enhanced expression and turnover of enzyme proteins (e.g., monooxygenase StyAB), which facilitates indigo synthesis. Second, enrichment in metabolic processes of reactive nitrogen species indicates a cellular response to oxidative stress induced by indigo biosynthesis. Given that indigo synthesis involves oxygenation reactions, regulating reactive nitrogen species helps maintain intracellular redox balance and reduce byproduct accumulation. Finally, enrichment in organophosphate ester transport may be necessary to provide energy for the transport and catabolism of tryptophan and tryptophan-based anabolism. KEGG pathway enrichment analysis (Figure 7B) further revealed significant enrichment in pathways related to nitrogen metabolism, amino acid biosynthesis, and membrane transport. Enrichment in nitrogen metabolism pathways suggests an enhanced capacity for tryptophan uptake and utilization, thereby facilitating the metabolic flux toward indigo biosynthesis. Enrichment in amino acid metabolic pathways (e.g., tryptophan and arginine) is directly linked to substrate accumulation, while enrichment in membrane transport pathways is consistent with the central roles of AcrA and Mtr in substrate uptake and product efflux. This suggests that tryptophan supplementation modulates the expression of these pathways, stimulating substrate uptake and product efflux to alleviate intracellular indigo toxicity and improve cellular vitality. Integrating GO and KEGG analyses reveals that indole supplementation achieves global metabolic network optimization (re-wiring) through coordinated regulation of protein metabolism, the oxidative stress response, and transport systems. The expression of key enzymes is enhanced to promote substrate uptake and indigo biosynthesis, while indigo efflux promotion alleviates intracellular indigo toxicity, allowing better substrate uptake and metabolism. This is consistent with the observed indigo yield increase in the engineered strains overexpressing *mtr* and *acrA*. This omics approach clarified the effects of the heterologous expression of genes involved in indigo biosynthesis on the metabolism of the *E. coli* host strain, providing useful information for developing engineered strains for industrial applications.

#### 3.2.3. Protein–Protein Interaction (PPI) Network and Module Analysis

**Figure 8:**
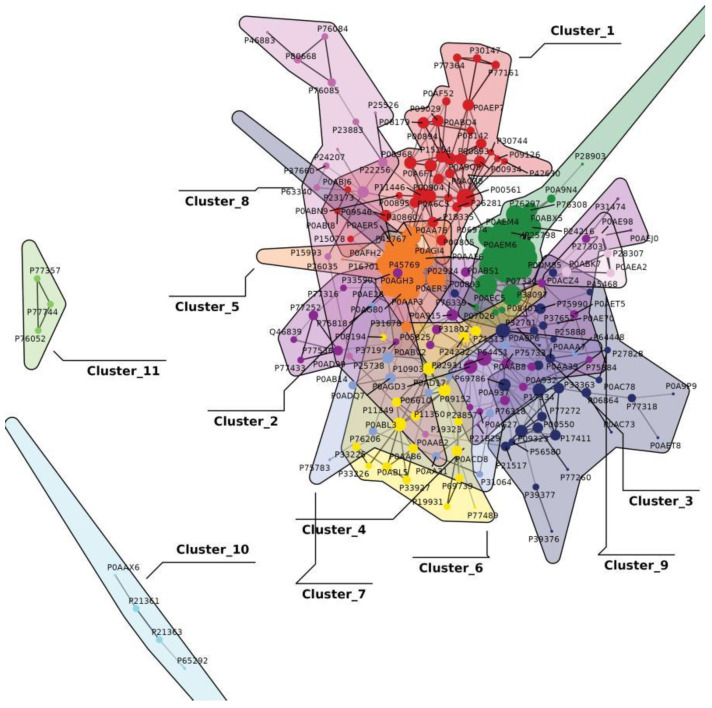
Protein–protein interaction (PPI) network of differentially expressed proteins in the E216-1 vs. E216-0 comparison. Interactions were retrieved from the STRING database (version 12.0) with a confidence score ≥ 0.700. Network visualization and module identification were performed using the igraph package in R (version 4.2.0). Eleven modules were identified based on clustering algorithms.

In the protein–protein interaction (PPI) network and module analysis (Figure 8), tryptophan addition was found to induce modular restructuring within the *E. coli* metabolic network, characterized by 11 functional clusters with dense intra-cluster interactions and sparse inter-cluster associations. This architecture reflects a highly specialized division of labor and coordination. The large-scale core metabolic module, enriched in nitrogen cycle, protein synthesis, and macromolecule metabolism, directly supports indigo synthesis by optimizing nitrogen source utilization and enzyme production (e.g., malate dehydrogenase, Mdh), consistent with the strategy of *mdh* overexpression to enhance cofactor regeneration. The medium-scale surface structure module, which included flagellar assembly and bio-film formation, enhances environmental adaptability and reduces physical damage from indigo in the extracellular medium during fermentation. The small-to-medium amino acid synthesis and transport module, enriched in arginine and lysine biosynthesis and organic anion transport, may contribute to pH homeostasis during fermentation, as the accumulation of tryptophan or indigo derivatives often causes medium acidification or alkalization; by maintaining an optimal pH environment, this module supports metabolic activity and tryptophan utilization, thereby indirectly boosting indigo yields. Finally, the small-scale stress response and detoxification module, enriched in chemical response and nitrotoluene degradation, mitigates tryptophan-derived toxicity and complements *acrA*-mediated efflux to maintain cellular vitality. This multi-module synergy—spanning core metabolism, surface construction, amino acid synthesis, and stress response—validates, at a systems level, how tryptophan addition coordinates substrate utilization, product efflux, and stress tolerance by activating a global PPI network. This finding provides molecular interaction-level evidence supporting the feasibility of multi-omics integration for green indigo production, particularly in strains overexpressing *acrA* and *mtr*. Notably, tryptophan catabolism pathways were not identified as enriched modules in the PPI network, consistent with the metabolic state under indigo-producing conditions.

## 4. Discussion

This study systematically investigated the global optimization effect of tryptophan supplementation on indigo biosynthesis via overexpression of the substrate transporter gene *mtr* and the indigo efflux gene *acrA* in the engineered *E. coli* strain E216. In addition, we conducted a comparative proteomic analysis of E216 grown in fermentation medium with or without tryptophan supplementation. Whereas the original strain E216 achieved yields of 170 mg/L at 30 °C and 120 mg/L at 37 °C, the Mtr overexpression strain E24 achieved a yield as high as 300 mg/L at 37 °C, demonstrating that Mtr permease ensures precursor supply by enhancing tryptophan transport efficiency. This demonstrates a substantial advancement compared to the results of recent studies, such as the 235 mg/L indigo titer achieved by Pham et al. (2024) [6] through an auto-inducible pathway, and highlights the superior efficiency of our transporter-focused engineering strategy. The AcrA overexpression strains E13 and E14 reached a yield of approximately 200 mg/L at 30 °C and 37 °C, confirming the core role of the AcrA efflux pump in alleviating intracellular toxicity caused by the accumulation of indigo and its toxic intermediate indole, as well as other metabolic stresses associated with tryptophan metabolism. *acrA* knockout consistently led to a significant decrease in indigo production, and the growth curves indicated enhanced vitality of the overexpression strains. Furthermore, the DIA-based comparative proteomic analysis of the engineered strain E216 grown in fermentation medium with or without tryptophan identified 113 upregulated and 348 downregulated proteins under conditions of tryptophan supplementation. The GO and KEGG enrichment analyses revealed that the activation of pathways related to protein metabolism, oxidative stress response, and membrane transport following tryptophan addition was consistent with the genetic engineering outcomes. Protein–protein interaction network analysis further unveiled the synergistic roles of 11 functional modules. For instance, the core metabolic module supported reducing power supply, and the stress response and detoxification module alleviated intracellular toxicity. This provides a molecular interaction-level explanation for how the metabolic network adjusts to tryptophan supplementation and indigo production through the modification of the level of expression of multiple pathways. Compared to existing studies, this innovative study reveals the positive impact of *acrA* and *mtr* genes on indigo production via multi-omics integration and complements previous studies on monooxygenase or cofactor engineering [34]. By deepening the integration of genetic and proteomic approaches, this study provides theoretical support for green indigo production and lays a foundation for systems biology research.

Despite the improved indigo yields observed in this study, the market price of tryptophan ($7.5–9.1/kg) and chemically synthesized indigo ($3.5–9.1/kg) highlights current economic challenges hindering microbial production. Future studies should focus on further increasing yields, utilizing low-cost substrates, and optimizing fermentation processes to improve cost competitiveness and facilitate industrial application. Finally, future work should expand gene target screening, incorporate dynamic multi-omics techniques to optimize fermentation, and advance industrial applications to offer new pathways for green bio-manufacturing.

## Figures and Tables

**Table 1:** Strains and plasmids used in this study.

Strains and Plasmids	Related Properties or Functions	Source
Strains		
*E. coli*-BL21 (DE3)	Used as host strain	Tiangen Biotech, Beijing, China
E216	*E. coli* BL21 (DE3) harboring pETPcat-*styAB*-P_T7_-*mdh*	Collection of Beijing Engineering and Technology Research Center of Food Additives
BL21-Cas9	*E. coli* BL21 (DE3) harboring pCas9	Constructed at Beijing Engineering and Technology Research Center of Food Additives
*acrA*-BL21-1/2/3/4/5 and *mtr*-BL21-1/2/3/4/5	*acrA* gene and *mtr* gene knockout strains constructed with various N20 sequences	Constructed at Beijing Engineering and Technology Research Center of Food Additives
BL21-*acrA*	BL21 derivative with the *acrA* gene knocked out	Constructed at Beijing Engineering and Technology Research Center of Food Additives
BL21-*mtr*	BL21 derivative with the *mtr* gene knocked out	Constructed at Beijing Engineering and Technology Research Center of Food Additives

## Data Availability

Data are contained within the article.

## Supplementary Materials

The following supporting information can be downloaded at: https://www.mdpi.com/article/10.3390/foods15081385/s1, Table S1: Primers used for PCR in this research.

## References

[1] Kupferschmidt K. (2019). In search of blue. Science.

[2] Manian A.P., Mueller S., Bechtold T., Pham T. (2023). Quantification of indigo on denim textiles as basis for jeans recycling. Dye. Pigment..

[3] Altunay N. (2021). An optimization approach for fast, simple and accurate determination of indigo-carmine in food samples. Spectrochim. Acta Part A-Mol. Biomol. Spectrosc..

[4] Pagnacco M.C., Maksimovic J.P., Nikolic N.T., Bogdanovic D.V.B., Kragovic M.M., Stojmenovic M.D., Blagojevic S.N., Sencanski J.V. (2022). Indigo Carmine in a Food Dye: Spectroscopic Characterization and Determining Its Micro-Concentration through the Clock Reaction. Molecules.

[5] Yin H., Chen H., Yan M., Li Z., Yang R., Li Y., Wang Y., Guan J., Mao H., Wang Y. (2021). Efficient Bioproduction of Indigo and Indirubin by Optimizing a Novel Terpenoid Cyclase XiaI in *E. coli*. Acs Omega.

[6] Pham N.N., Wu Y.-H., Dai T.-A., Tu J., Liang R.-M., Hsieh H.-Y., Chang C.-W., Hu Y.-C. (2024). Auto-inducible synthetic pathway in *E. coli* enhanced sustainable indigo production from glucose. Metab. Eng..

[7] Wang W., Wu Y., Xu H., Shang Y., Chen Y., Yan M., Li Z., Walt D.R. (2019). Accumulation mechanism of indigo and indirubin in Polygonum tinctorium revealed by metabolite and transcriptome analysis. Ind. Crops Prod..

[8] Lopes H.d.F.S., Tu Z., Sumi H., Yumoto I. (2021). Analysis of bacterial flora of indigo fermentation fluids utilizing composted indigo leaves (*sukumo*) and indigo extracted from plants (Ryukyu-ai and Indian indigo). J. Biosci. Bioeng..

[9] Ahn S., Park S., Kumar P., Choi K.-Y. (2023). Bio-indigo Production using Wild-type Acinetobacter sp. and Indole-3-acetate Monooxygenase (iacA) Expressed in *E. coli*. Biotechnol. Bioprocess Eng..

[10] Tu Z., Lopes H.d.F.S., Igarashi K., Yumoto I. (2019). Characterization of the microbiota in long- and short-term natural indigo fermentation. J. Ind. Microbiol. Biotechnol..

[11] Fabara A.N., Fraaije M.W. (2020). An overview of microbial indigo-forming enzymes. Appl. Microbiol. Biotechnol..

[12] Schweiggert R.M. (2018). Perspective on the Ongoing Replacement of Artificial and Animal-Based Dyes with Alternative Natural Pigments in Foods and Beverages. J. Agric. Food Chem..

[13] Zhijie Q., Xinglong W., Song G., Dong L., Jingwen Z. (2023). Production of natural pigments using microorganisms. J. Agric. Food Chem..

[14] Li Y., Lin Y., Wang F., Wang J., Shoji O., Xu J. (2023). Construction of Biocatalysts Using the P450 Scaffold for the Synthesis of Indigo from Indole. Int. J. Mol. Sci..

[15] Kim J., Lee P.-G., Jung E.-O., Kim B.-G. (2018). In vitro characterization of CYP102G4 from Streptomyces cattleya: A self-sufficient P450 naturally producing indigo. Biochim. Biophys. Acta-Proteins Proteom..

[16] van Hellemond E.W., Janssen D.B., Fraaije M.W. (2007). Discovery of a novel styrene monooxygenase originating from the metagenome. Appl. Environ. Microbiol..

[17] Rosano G.L., Morales E.S., Ceccarelli E.A. (2019). New tools for recombinant protein production in *E. coli*: A 5-year update. Protein Sci..

[18] Gong Z., Wang H., Tang J., Bi C., Li Q., Zhang X. (2020). Coordinated Expression of Astaxanthin Biosynthesis Genes for Improved Astaxanthin Production in *E. coli*. J. Agric. Food Chem..

[19] Sorensen H.P., Mortensen K.K. (2005). Advanced genetic strategies for recombinant protein expression in *E. coli*. J. Biotechnol..

[20] Hou B., Song J., Wang H., Ye N., Wang R.-W. (2024). Tryptophan Transport Gene Inactivation Promotes the Development of Antibiotic Resistance in *Escherichia coli*. FEMS Microbiol. Lett..

[21] Ahmed M.S., Lauersen K.J., Ikram S., Li C. (2021). Efflux Transporters’ Engineering and Their Application in Microbial Production of Heterologous Metabolites. Acs Synth. Biol..

[22] Van Dyk T.K., Templeton L.J., Cantera K.A., Sharpe P.L., Sariaslani F.S. (2004). Characterization of the *Escherichia coli* AaeAB Efflux Pump: A Metabolic Relief Valve?. J. Bacteriol..

[23] Zhao J., Fang H., Zhang D. (2020). Expanding Application of CRISPR-Cas9 System in Microorganisms. Synth. Syst. Biotechnol..

[24] Tsushima A., Gan P., Kumakura N., Narusaka M., Takano Y., Narusaka Y., Shirasu K. (2019). Genomic Plasticity Mediated by Transposable Elements in the Plant Pathogenic Fungus *Colletotrichum higginsianum*. Genome Biol. Evol..

[25] Dalvie N.C., Lorgeree T., Biedermann A.M., Love K.R., Love J.C. (2022). Simplified Gene Knockout by CRISPR-Cas9-Induced Homologous Recombination. Acs Synth. Biol..

[26] Zijing P., Dejiang T., Mingjing R., Lei C. (2023). A Combinational Optimization Method for Efficient Production of Indigo by the Recombinant *Escherichia coli* with Expression of Monooxygenase and Malate Dehydrogenase. Foods.

[27] Funakoshi Y., Azuma A., Ishikawa M., Itsuki S., Tamura Y., Kanemaru K., Hirai S., Oyama Y. (2018). Cytometrical Analysis of the Adverse Effects of Indican, Indoxyl, Indigo, and Indirubin on Rat Thymic Lymphocytes. Toxicol. Res..

[28] Wang Y., He Y., Qian X., Zheng X., Wang Y., Gong Q. (2025). Exploring Diversity of Conopeptides and Revealing Novel Conoinsulins from Conus Betulinus by Proteomic Analyses. J. Proteome Res..

[29] Wallmann G., Skowronek P., Brennsteiner V., Lebedev M., Thielert M., Steigerwald S., Kotb M., Despard O., Heymann T., Zhou X.-X. (2025). AlphaDIA Enables DIA Transfer Learning for Feature-Free Proteomics. Nat. Biotechnol..

[30] Guo T., Aebersold R. (2023). Recent Advances of Data-Independent Acquisition Mass Spectrometry-based Proteomics. Proteomics.

[31] Brazelton V.A., Zarecor S., Wright D.A., Wang Y., Liu J., Chen K., Yang B., Lawrence-Dill C.J. (2015). A quick guide to CRISPR sgRNA design tools. GM Crops Food-Biotechnol. Agric. Food Chain.

[32] Gotz S., Garcia-Gomez J.M., Terol J., Williams T.D., Nagaraj S.H., Nueda M.J., Robles M., Talon M., Dopazo J., Conesa A. (2008). High-Throughput Functional Annotation and Data Mining with the Blast2GO Suite. Nucleic Acids Res..

[33] Kanehisa M., Goto S., Sato Y., Furumichi M., Tanabe M. (2012). KEGG for Integration and Interpretation of Large-Scale Molecular Data Sets. Nucleic Acids Res..

[34] Chen T., Wang X., Zhuang L., Shao A., Lu Y., Zhang H. (2021). Development and Optimization of a Microbial Co-Culture System for Heterologous Indigo Biosynthesis. Microb. Cell Factories.

