# Supplementary material for: Insights into the Regulation of Indigo Production in an Engineered Escherichia coli Strain via Overexpression of Specific Transporter Genes and Proteomic Analyzes
Source: Foods. 2026 Apr 16;15(8):1385. doi: 10.3390/foods15081385 (PMC13115177; doi:10.3390/foods15081385)
Supplement: Supplementary file 1 [file foods-15-01385-s001.zip › Supplementary.pdf]

Article

Mechanistic Insights into Optimizing Indigo Production in Engineered *Escherichia coli* Through Gene Regulatory and Proteomic Analyses

Jie Gao [1], Anni Fang[1], Tianjiao Meng [1], Baoguo Sun [1], and Lei Cheng [1,*]

[1] Key Laboratory of Plant Protein Innovation and Resource Development, China National Light Industry, Beijing Engineering and Technology Research Center of Food Additives, Beijing Technology and Business University, Beijing 100048, China; jiegao0220@163.com(J.G.); 1363785062@qq.com(A.F.); mtianjiao0716@163.com(T.M.); sunbg@btbu.edu.cn(B.S.);

* Correspondence: chenglei@btbu.edu.cn

Table S1. Primers used for PCR in this research

Primer	Sequence (5′-3′)
acrA-F	ATGCAGACCCCGCACATTCTTATC
acrA-R	TTAATCTTCCAGATCACCGCAGAAG
mtr-F	ATGGCAACACTAACCACCACCC
mtr-R	TTACTGATACACCGGCAGTAAATTA
pTargetF-line-F	TCGAGTTCATGTGCAGCTCC
pTargetF-line-R	GTAGGGATAACAGGGTAATA
acrA-1-F	TTCAACGATAAGAATGTGCGACTAGTATTATACCTAGGAC
acrA-1-R	CGCACATTCTTATCGTTGAAGTTTTAGAGCTAGAAATAGC
acrA-2-F	CTTCAACGATAAGAATGTGCACTAGTATTATACCTAGGAC
acrA-2-R	GCACATTCTTATCGTTGAAGGTTTTAGAGCTAGAAATAGC
acrA-3-F	TCTTCAACGATAAGAATGTGACTAGTATTATACCTAGGAC
acrA-3-R	CACATTCTTATCGTTGAAGAGTTTTAGAGCTAGAAATAGC
acrA-4-F	TCTTATCGTTGAAGACGAGTGTTTTAGAGCTAGAAATAGC
acrA-4-R	ACTCGTCTTCAACGATAAGAACTAGTATTATACCTAGGAC
acrA-5-F	TTTCGAAGCGACAGATGGCGGTTTTAGAGCTAGAAATAGC
acrA-5-R	CGCCATCTGTCGCTTCGAAAACTAGTATTATACCTAGGAC
CRI-*acrA*-F	ACCGAGTCGGTGCTTTTTTTGGTACCGGCGATCTTTCTGTGG
CRI-*acrA*-R	GGGATAACAGGGTAATTCGTTAGTCAGCCCGGAAT
mtr-1-F	CGACGGTGACGTTTGGGTGGACTAGTATTATACCTAGGAC
mtr-1-R	CCACCCAAACGTCACCGTCGGTTTTAGAGCTAGAAATAGC
mtr-2-F	CAGCGACGGTGACGTTTGGGACTAGTATTATACCTAGGAC
mtr-2-R	CCCAAACGTCACCGTCGCTGGTTTTAGAGCTAGAAATAGC
mtr-3-F	ACCACGCCGCCAAGCAGCGAACTAGTATTATACCTAGGAC
mtr-3-R	TCGCTGCTTGGCGGCGTGGTGTTTTAGAGCTAGAAATAGC
mtr-4-F	CAAACGTCACCGTCGCTGCTGTTTTAGAGCTAGAAATAGC
mtr-4-R	AGCAGCGACGGTGACGTTTGGTTTTAGAGCTAGAAATAGC
mtr-5-F	ACGTCACCGTCGCTGCTTGGGTTTTAGAGCTAGAAATAGC
mtr-5-R	CCAAGCAGCGACGGTGACGTACTAGTATTATACCTAGGAC
CRI-*mtr*-F	ACCGAGTCGGTGCTTTTTTTGGCTGGGTCGTGCTCCGGGT
CRI-*mtr*-R	GTAGGGATAACAGGGTAATCGTTGAGAACCGCGAGCGTC
over-28a-F	ATTGCGCCCAGCGCCATCTG

over-28a-R	CACCATCAAACAGGATTTTC
F-*acrA*-over	GCGCCGAGACAGAACTTAATATGCAGACCCCGCACATTCT
R-*acrA*-over	TGGAGCATCTGGTCGCATTGTTAATCTTCCAGATCACCGC
F-*mtr*-over	GCGCCGAGACAGAACTTAATATGGCAACACTAACCACCAC
R-*mtr*-over	TGGAGCATCTGGTCGCATTGTTACTGATACACCGGCAGTA
T_7+*acrA*-F	GAGCGGATAACAATTCCCCAAGGAGGAACTATATCCGGAT
T_7+*acrA*-R	TTAATCTTCCAGATCACCGCAGAAG
T7+*mtr*-F	TAATACGACTCACTATAGGGATGGCAACACTAACCACCAC
T_7+*mtr*-R	TTACTGATACACCGGCAGTAAATTA
over-T_7-*acrA*-F	GAGCGGATAACAATTCCCCATGCAGACCCCGCACATTCT
over-T_7-*acrA*-R	CATCTGGTCGCATTGTTAATCTTCCAGATCACCGC
over-T_7-*mtr*-F	GAGCGGATAACAATTCCCCATGCAGACCCCGCACATTCT
over-T_7-*mtr*-R	TGGAGCATCTGGTCGCATTGTTACTGATACACCGGCAGTA
